# Rate Effects on Timing, Key Velocity, and Finger Kinematics in Piano Performance

**DOI:** 10.1371/journal.pone.0020518

**Published:** 2011-06-23

**Authors:** Simone Dalla Bella, Caroline Palmer

**Affiliations:** 1 Department of Cognitive Psychology, University of Finance and Management, Warsaw, Poland; 2 Department of Psychology, McGill University, Montreal, Canada; The University of Western Ontario, Canada

## Abstract

We examined the effect of rate on finger kinematics in goal-directed actions of pianists. In addition, we evaluated whether movement kinematics can be treated as an indicator of personal identity. Pianists' finger movements were recorded with a motion capture system while they performed melodies from memory at different rates. Pianists' peak finger heights above the keys preceding keystrokes increased as tempo increased, and were attained about one tone before keypress. These rate effects were not simply due to a strategy to increase key velocity (associated with tone intensity) of the corresponding keystroke. Greater finger heights may compensate via greater tactile feedback for a speed-accuracy tradeoff that underlies the tendency toward larger temporal variability at faster tempi. This would allow pianists to maintain high temporal accuracy when playing at fast rates. In addition, finger velocity and accelerations as pianists' fingers approached keys were sufficiently unique to allow pianists' identification with a neural-network classifier. Classification success was higher in pianists with more extensive musical training. Pianists' movement “signatures” may reflect unique goal-directed movement kinematic patterns, leading to individualistic sound.

## Introduction

The production of auditory sequences such as music and speech is generally fluent and accurate. One hallmark of fluency is the performer's ability to prepare for upcoming events. As musicians' performance skill increases, their ability to anticipate upcoming sequence events increases, as evidenced in performance errors [1], [2]. Skilled movements show evidence of preparation as well. This can be observed in coarticulation effects in speech: contextual influences of upcoming sequence elements on the production of current elements, which aids the fluent production of movement sequences [3], [4], [5]. Study of movement kinematics is likely to shed light on motor and cognitive constraints underlying music performance [5], [6], and may distinguish among skill levels. There are few studies devoted to movement kinematics in music performance in relation to the sounded outcome of performance. The current study investigates relationships between pianists' finger movements and characteristics of the resulting sound (timing and intensity of tones) in performance.

Pianists' fingers can approach their instruments by following many different trajectories (the degrees of freedom problem) [7]. At the same time, finger movements are subject to constraints of fine spatial and temporal control, which require performers to produce correct pitches, accurate timing, and an intended expression. Cognitive constraints affect fluent performance as well; individual differences in working memory constrain how many and which pitch events can be anticipated in action planning prior to production [2], [8], [9]. Biomechanical factors, such as the degree of independence between fingers [10], also provide constraints on the range of possible movements [11], [12]. Thus, finger movements in piano performance are likely to reflect both cognitive and biomechanical constraints [5], [6].

There are few studies of finger movement in piano performance. Although the timing of hammer-string interaction in acoustic grand piano mechanisms is well-documented [13], [14], [15], most quantitative studies of piano performance, based on computer-monitored MIDI instruments, do not measure performers' movements. The recent advent of motion capture techniques based on passive (non-interfering) markers has made it feasible to measure motion in music performance. Using motion capture, Engel et al. [3] recorded movements of pianists' right wrist and fingers while they performed musical pieces that contained identical beginnings but different continuations. Anticipatory movement, measured by the divergence of finger movement trajectories, occurred as much as 500 ms in advance of the last note shared by the melodies. More recently, Loehr and Palmer [5] documented with motion capture that pianists' finger tapping movements reached maximum height above the tabletop about 200 ms before each tap (approximately one-half of the intertap intervals in that study). They found that biomechanical constraints (finger coupling), more than cognitive constraints (chunking), affected the kinematics of the finger movements (i.e., velocity and acceleration trajectories). In a further experiment in which pianists tapped at different rates [6],finger motions that were preceded by a more coupled finger's tap showed larger timing error and change in motion due to the preceding finger. This indicates that timing of sequence elements is not independent of the motion trajectories used to produce them. Effects of movement properties on the timing of subsequent events, and in particular on tactile feedback has been recently documented in piano performance [16]. Using a synchronization-continuation paradigm, pianists' finger movements were captured during performance of simple melodies at different rates. Increased tactile information as fingers reached piano keys (based on kinematic measures of acceleration peaks in finger trajectories) was related to increased temporal accuracy of the upcoming keystroke. In sum, these studies suggest that there may be a tight relationship between fingers' motion and temporal properties of piano performance.

The principal aim of the present study was to examine the effects of performance tempo on pianists' finger movements and on properties of the sounded tones (tone intensity and temporal variability). Pianists tend to play louder and with greater temporal variability at faster tempi [17], [18], [19], [20], but it is not known how finger movements bring about these effects. Interestingly, music-pedagogical techniques usually promote a principle of economy of finger movement on various instruments [21], [22], [23], in which performers are encouraged to keep their fingers close to the keys during fast passages in order to conserve energy. Studies showing that faster rates of finger movements in the air (with no contact) yielded smaller-amplitude movements in two-finger oscillation tasks [24] confirmed this relationship between speed and movement amplitude. Finally, Palmer and Pfordresher's memory retrieval model [2] predicts effects of tempo on movement preparation. Although their model does not make explicit predictions for finger movements, the finding that memory for a larger range of sequence events is available during performance at slower tempi suggests that movements should be prepared sooner at slower tempi than at fast tempi [25]. In sum, based on pedagogical theories, models of interlimb coordination, and models of memory retrieval, we hypothesized that pianists' finger movements would have smaller movement amplitudes and show less anticipation at fast tempi than at slow tempi.

An additional goal was to test whether the kinematics of pianists' finger movement associated with striking and releasing piano keys are individualistic. Personal identification based on properties of biological motion is revealed, for example, in studies on gait with the point-light display technique [26], [27]. It is noteworthy that gait kinematics alone (velocity and acceleration patterns), when structural information (e.g., size) and walking frequency are kept constant, yields more than 80% correct identification of individuals [28], [29]. In the auditory modality, it is likely that kinematic features of pianists' finger movements similarly give rise to individuated sound in performance, thereby reflecting artists' musical uniqueness. Note that some biomechanical constraints, such as finger coupling, are largely shared by individuals [5], [10]. For example, pianists' individual finger movements are often accompanied by movements of neighboring (physically adjacent) fingers due to biomechanical constraints [5]. We propose that performers may achieve spatial and temporal accuracy, two essential goals in piano performance, by adopting different movement strategies that in turn yield different sound outcomes. The possibility that the uniqueness of a given pianist's style is tied to idiosyncratic kinematic properties of finger movement has not been examined thus far.

In this exploratory study, four skilled pianists with different levels of musical expertise performed short melodies on an electronic keyboard at different tempi while their finger movements were recorded with motion capture techniques. We contrasted pianists' movements as fingers approached and stayed on the keys with the same finger movements when they were “at rest” (while other fingers struck keys). In particular, we examined the relationship between the properties of fingers' movement (amplitude and time of peak height) in the vertical direction (which affect how strongly keys are struck and how loud the resulting sound is) and their implications for the outcome of the performance (timing and intensity of tones). In addition, we tested whether kinematic properties of anticipatory movements are sufficient to identify the pianists by training and testing a neural network classifier on different time segments of the finger trajectories. We predict that the pianists may adopt different movement strategies when playing at different tempi, based on their levels of musical expertise.

## Results

### Timing analyses

**Figure 1:**
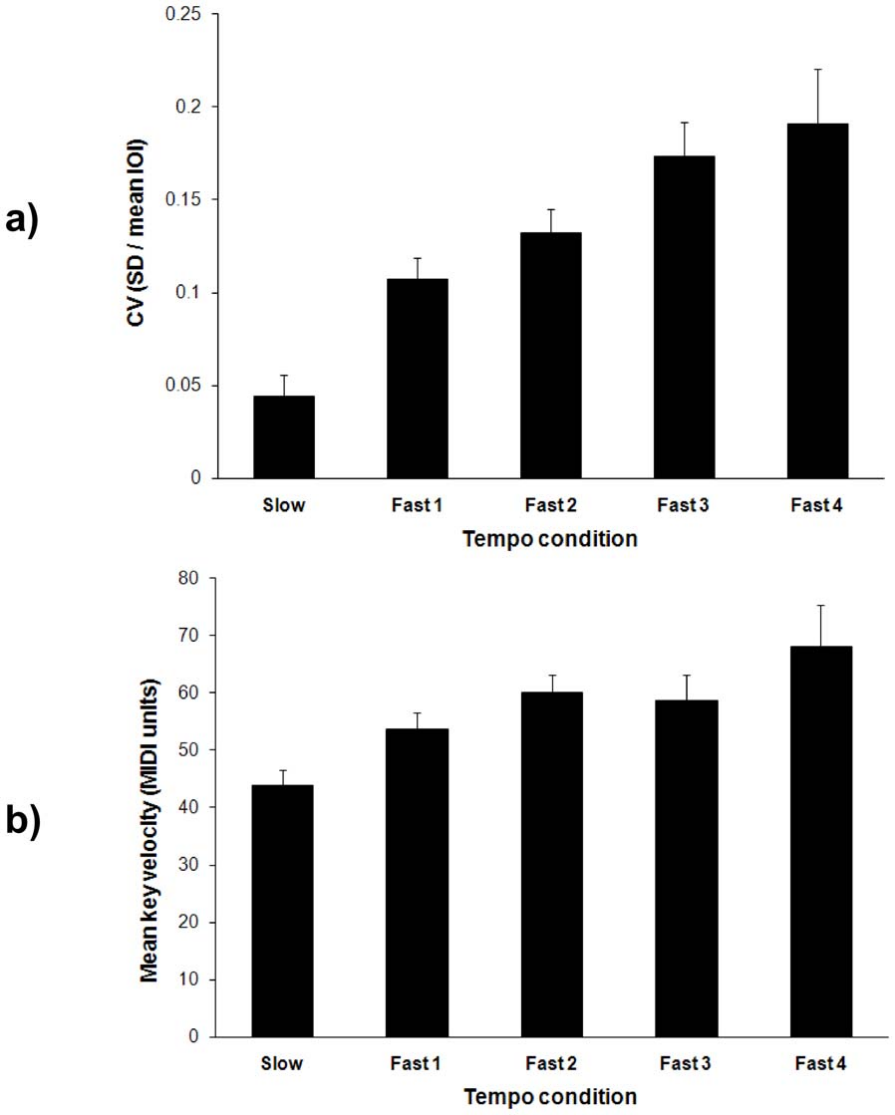
Results from MIDI analyses. a) Mean coefficients of variation (CV) of the IOIs (SD/mean IOI) for the five tempo conditions; b) Mean key velocity (tones' intensity) for the five tempo conditions. Error bars are SE of the Mean.

The eighth-note interonset intervals (IOIs) associated with each tone were computed from the keyboard MIDI data. The first and last tones of each performance were excluded from both MIDI and movement analyses because they did not have comparable beginnings or endings, respectively. The pianists were very accurate in performing at each prescribed tempo: the mean IOIs were on average within 4% (7 ms) of the prescribed IOIs. Accuracy in performing at the prescribed tempo, measured in percentage deviation from expected values ((|observed mean IOI - expected IOI|/expected IOI)*100) changed as tempo increased (Slow, 0.6%; Fast1, 4.7%; Fast2, 7.2%; Fast3, 1.5%; Fast4, 2.2%; *F*(4,40) = 2.63, *p*<.05). Because not all pianists were capable of reaching the error-free performance criterion at the fastest tempi, performance was treated as the random variable to avoid missing cases. To confirm that the tempo effects yielded by the ANOVA were not confounded with individual differences, the effect of tempo was tested in each pianist separately with Kruskal-Wallis non-parametric tests. Post-hoc comparisons revealed that this effect was mostly due to a significant difference between Fast1 and Fast3 tempi (Tukey HSD, *p*<.05). Pianists' note timing relative to the mean IOI was also more variable at faster tempi, as shown by the mean coefficient of variation (CV = *SD* divided by the mean IOI). Figure 1(a) shows the mean CVs, computed for each performance and averaged across tempi and participants, which increased with tempo (*F* (4, 40) = 13.9, *p*<.001). Thus, relative temporal precision decreased as tempo increased. In addition, as shown in Figure 1(b), MIDI key velocities indicated that tones' intensity increased as tempo increased (*F* (4, 40) = 5.3, *p*<.01). These effects of tempo on both CVs and key velocities were observed in all pianists, as indicated by non-parametric Kruskal-Wallis tests (for Pianists 2–4, *p*<.05; Pianist 1, *p* = .07, marginally significant).

### Movement analyses

Pianists' finger movements were examined in the vertical (height) dimension perpendicular to the horizontal plane of the piano keyboard. Motion data from the fingertip markers only were analyzed. The discrete data from finger positions were converted into continuous functions with Functional Data Analyses (FDA) [30]. The position values were smoothed with order-6 splines as basis functions with a smoothing parameter (*lambda*) between 10^−12^ and 10^−13^, which smoothes the acceleration (second derivative) curves. These parameter values yielded fits which fell within .00001 of the generalized cross-validation score that estimates the optimal degree of smoothing by minimizing the expected mean square error between raw and fitted data [31].

**Figure 2:**
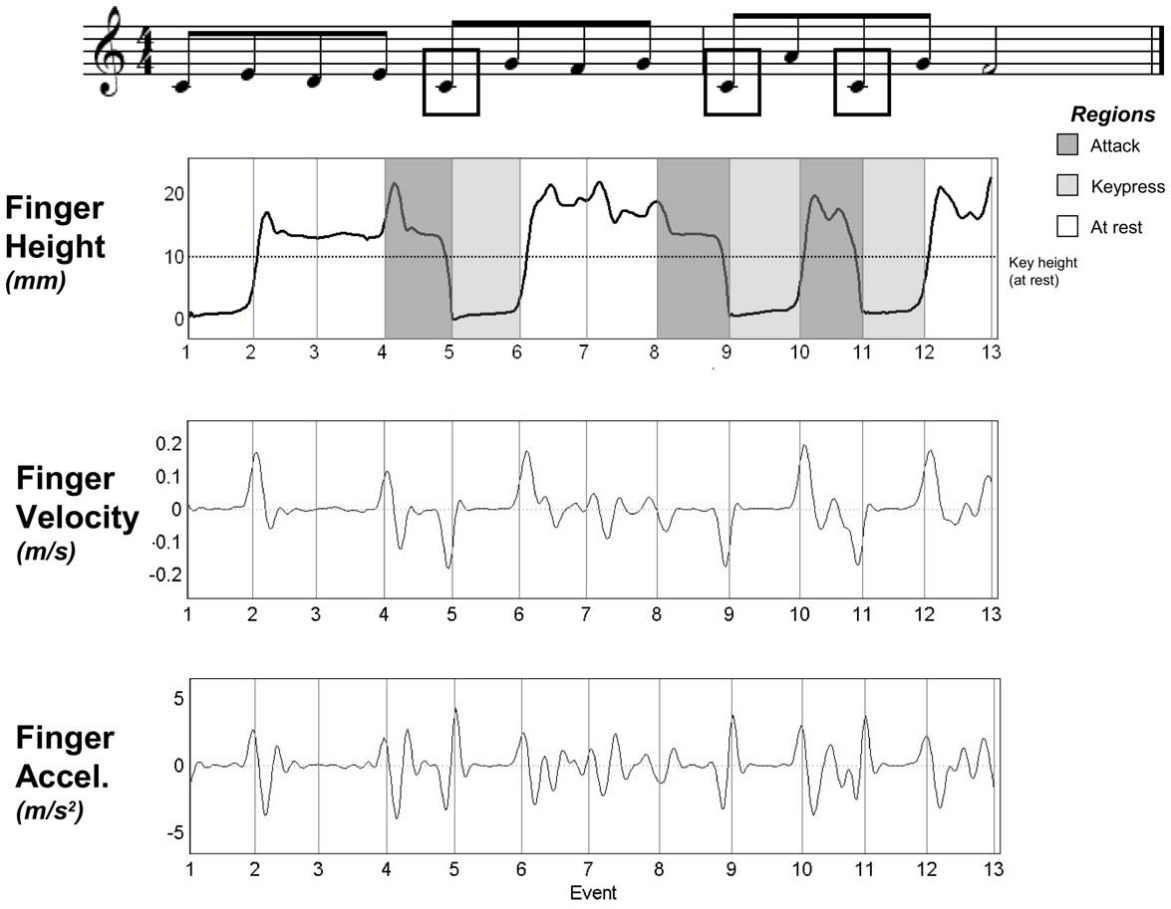
Movement of Finger 1 (thumb) during one pianist's performance of the notated melody at Slow tempo. Framed notes represent keypresses for Finger 1; vertical lines indicate the time at which the piano keys reached the bottom for each note in the melody. Highlighted regions underscore motion *attack* regions (dark gray areas) and *keypress* regions (light gray areas). Key height at rest was approximately 10 mm (+/− 0.5 mm).

Figure 2 shows the smoothed position, velocity, and acceleration curves for one pianist's thumb movements (Finger 1) during a performance at the Slow tempo. The markers on the piano keys served to identify the time of keypress. The vertical lines in Figure 2 denote the minimum position values of the piano key markers, which indicate the time at which the piano key reached key bottom (within one sample or 8 ms of the MIDI keypress event). Keypresses were aligned across performances in terms of the key bottom positions; the region between two successive vertical lines is referred to as an *event region*. Dark grey shading indicates an event region preceding a keypress (hereafter referred to as ‘attack regions’) and light gray shading indicates the region during a keypress (‘keypress region’). Positive and negative velocities indicate movement away and toward the key, respectively.

**Figure 3:**
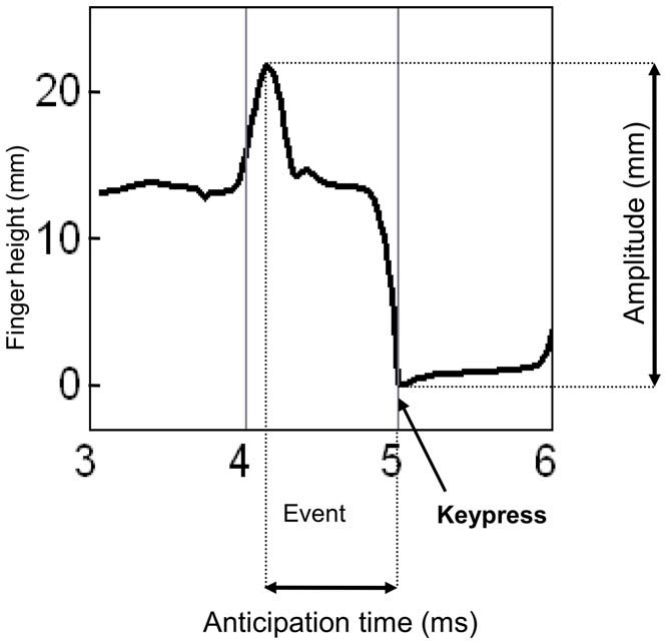
Movement amplitude and anticipation time for a portion of the finger height trajectory framed in **Figure 2**.

The *finger movement amplitude*, computed over two event regions prior to the keypress, is defined here as the difference (in mm) between the maximum finger height before keypress and the minimum finger height at keypress (defined as 0 height). *Anticipation time* before keypress is defined as the difference (in ms) between the time of the maximum finger height within the two event regions prior to the keypress, and the time of the keypress (defined as time 0). Figure 3 illustrates the computation of movement amplitude and anticipation time for a portion of the finger height trajectory presented in Figure 2. The shorter the anticipation time, the closer the peak movement amplitude is to the keypress. Trajectories in which the finger was resting on the key at maximum finger height were excluded from amplitude and anticipation time analyses; only cases in which the finger was raised above the key were included (80% of all trajectories). Cases of fingers resting on the key prior to keypress were observed in all pianists. This phenomenon concerned primarily enslaved fingers [5], such as the middle finger and the ring finger (64% of on-key trajectories), which are most constrained by neighboring fingers during performance. This indicates that biomechanical constraints affected finger movement amplitude to some extent.

**Figure 4:**
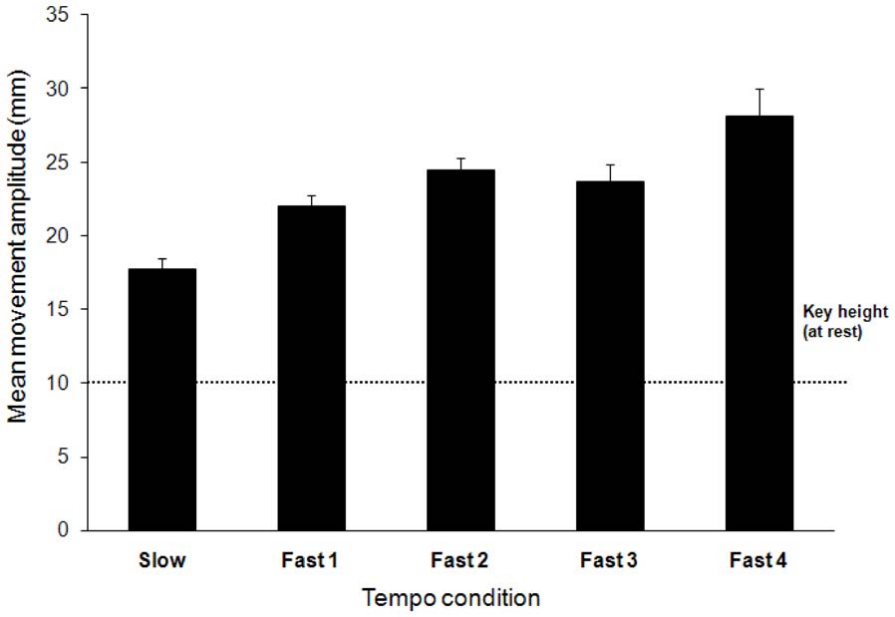
Mean movement amplitude averaged across all fingers for the five tempo conditions. Errors bars indicate standard errors. Key height at rest was approximately 10 mm (+/− 0.5 mm).

Pianists' mean finger movement amplitudes, averaged across all fingers, are shown in Figure 4 by tempo condition. Pianists lifted fingers higher as tempo increased (*F* (4, 40) = 13.87, *p*<.001). Post-hoc tests (Tukey HSD) revealed that the Slow tempo significantly differed from all other tempi (*p*<.01); in addition, Fast1 significantly differed from Fast4 (*p*<.05). Increasing tempo led to larger finger movement amplitudes in all pianists (for Pianists 2–4, *p*<.05; Pianist 1, *p* = .07, marginally significant). As expected, movement amplitudes differed among fingers, with the thumb amplitude reaching a maximum value (26.1 mm on average), and the ring finger reaching a minimum value (21.7 mm) (*F* (4, 92) = 2.97, *p*<.05). Differences in finger heights are expected, given the different lengths and degrees of freedom of different fingers [5]. There was no interaction of tempo with finger; all finger movement amplitudes increased with tempo.

We compared finger movement amplitudes with the temporal and intensity characteristics of the sounded tones. Larger finger movement amplitudes were associated with increases in MIDI key velocity (*r* = .73, *p*<.01). To investigate whether tempo or tone intensity goals accounted for increased finger heights at faster tempi, the effects of key velocity were first partialled out from finger movements by regressing key velocity on movement amplitudes for each finger movement. The effect of tempo on residual finger amplitudes was still significant (*F* (4,40) = 3.17, *p*<.05), indicating that tempo goals contributed to finger heights beyond intensity goals.

The mean anticipation times (ms before key bottom that the finger reached maximum amplitude) across fingers were larger at the slower tempi (*F* (4, 40) = 72.92, *p*<.001), an effect that was observed in all pianists (for Pianists 2–4, *p*<.05; Pianist 1, *p* = .07, marginally significant). The mean anticipation time was equivalent to about one tone before the keypress across tempo conditions. This is confirmed by the analysis of anticipation times expressed in % of the IOI. Relative anticipation times did not differ significantly as a function of tempo (Slow = 111% of the IOI, Fast1 = 107%, Fast2 = 100%, Fast3 = 113%, and Fast4 = 123%). The Slow tempo differed from all other tempi, as indicated by post-hoc tests (Tukey HSD, *p*<.01). Anticipation time was negatively correlated with MIDI key velocity (*r* = −.47, *p*<.01): the shorter the anticipation time, the louder the tone. To assess whether intensity changes were associated with tempo effects on anticipation times, the effects of key velocity on anticipation times were partialled out, as before; the effect of tempo on residual finger anticipation times was still highly significant (*F* (4,40) = 16.10, *p*<.001). Thus, finger movements in anticipation of a keypress started sooner at slower tempi, above and beyond intensity changes.

**Figure 5:**
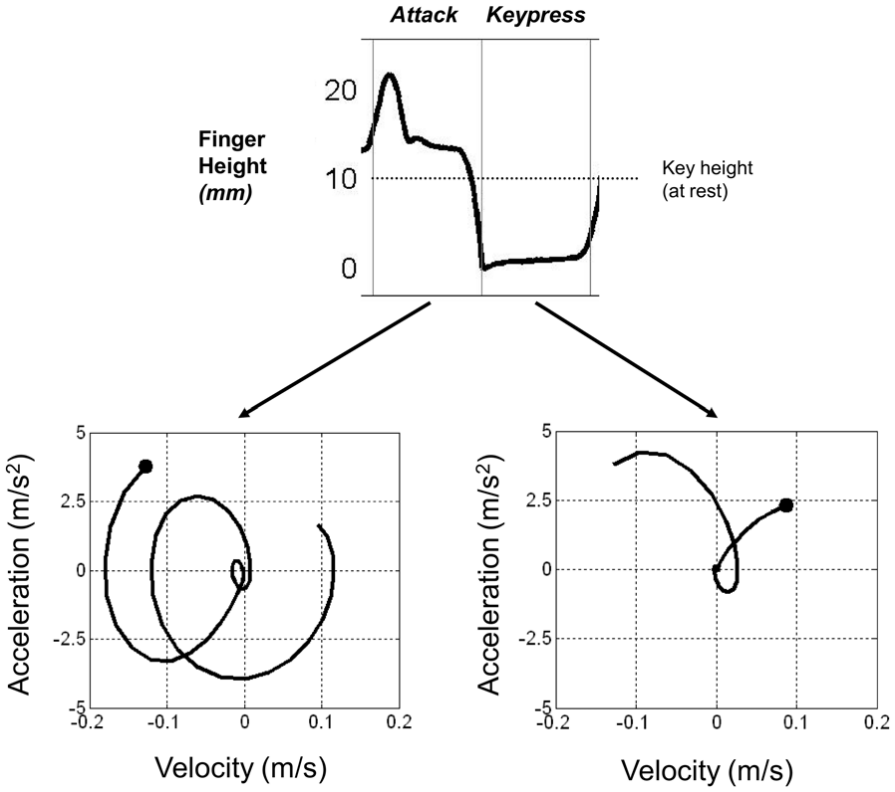
Finger height and phase plane plots of velocity-acceleration trajectory for a pianist's Finger 1 (thumb) movement in the *attack* and *keypress* event region (see **Figure 2**). Black filled circles mark the end of movement, corresponding to full key pressure.

Each finger's movement kinematics one event before and during keypresses was compared with its kinematic properties while other fingers were pressing keys. Figure 5 shows an example of a velocity-acceleration trajectory for Finger 1 (the thumb) for the *attack* region (lower left panel) and for the *keypress* region (lower right-panel). We examined whether the consistency of kinematic properties across movements by the same finger varies depending on the type of event region. Each velocity-acceleration finger trajectory was compared with other trajectories; the similarity of the trajectories was computed between all possible pairs (*n* = 496, without pairs including the same trajectories) for *attack*, *keypress*, and remaining event regions, termed “at-rest” (while another finger struck a key). Similarity was computed with Procrustean transformation methods [32] which estimate a linear transformation of the points in one matrix to best conform to the points in another matrix. Procrustes values range from 0 (no shape similarity) to 1 (identical shape). Both the *attack* finger movements (mean Procrustes value = .32) and the *keypress* finger movements (mean = .47) were significantly more consistent than movements *at rest* (mean = .17, *F* (2,477) = 77.9, *p*<.01, taking trajectory as the random factor). Thus, finger movements were more consistent during the goal of striking a key.

### Network Classification Analyses

**Figure 6:**
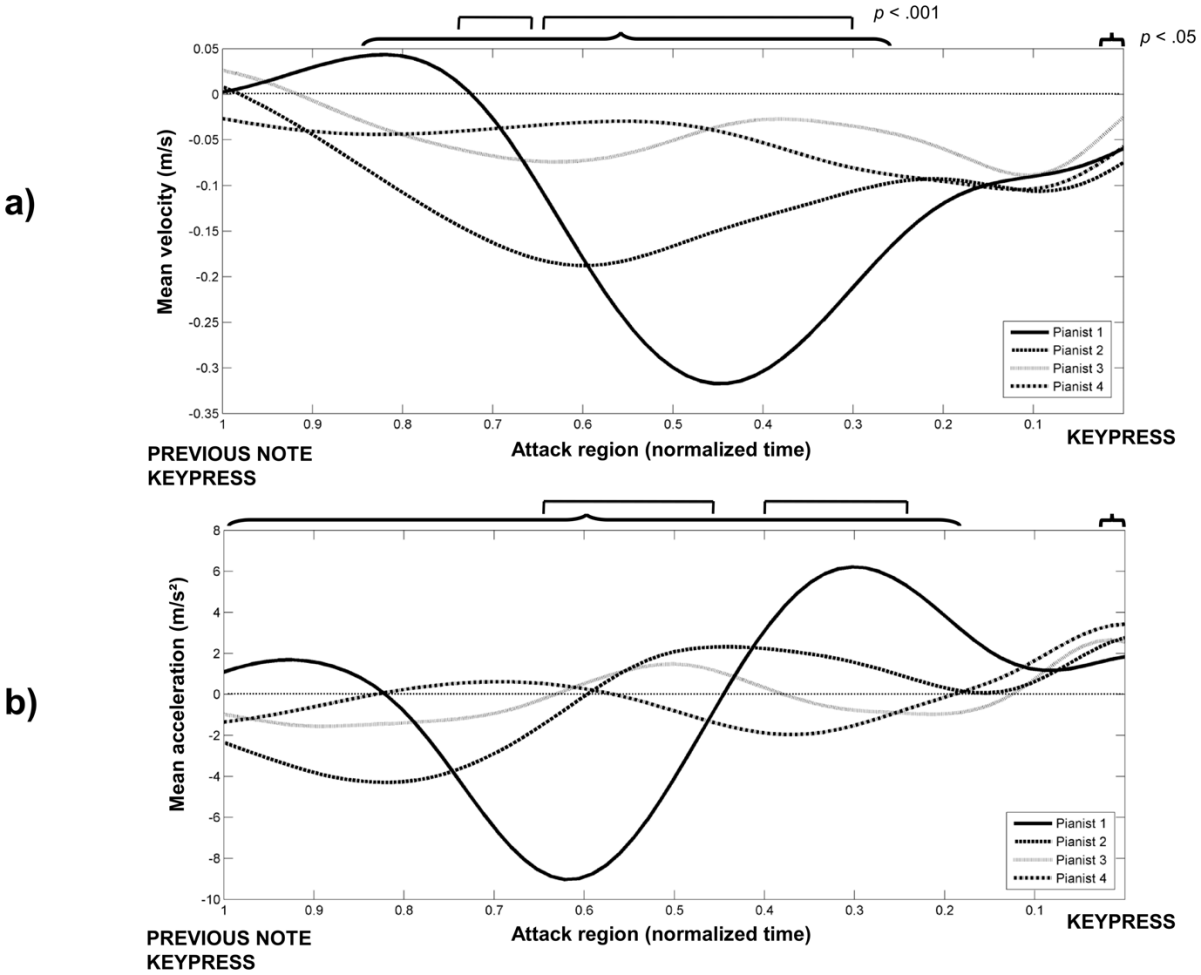
Mean velocity (panel *a*) and acceleration (panel *b*) trajectories for Finger 2 (index) in the *attack* event region for the 4 performers. Brackets indicate the regions where the difference between performers reached significance (with *p*<.05, and *p*<.001 significance values). The x-axis is the normalized time in the *attack* event region: 1 refers to the keypress of the preceding note (i.e., beginning of the *attack* region) and 0 to time of occurrence of the actual keypress.

Next we tested whether the movement trajectories for *attacks*, *keypresses*, and *at-rest* event regions contained sufficient information to identify each performer on the basis of individual keystrokes. The portion of the velocity-acceleration curves for each finger that differed significantly between pianists was first identified by Functional ANOVAs [30], which allow comparisons among functional observations using a functional linear model. Figure 6 shows the mean velocity and acceleration trajectories (panels *a* and *b*, respectively) for the index finger across tempo conditions in the *attack* event region for each of the performers. The brackets along the top of each panel indicate the portion of the event region (69%) in which the main effect of Performer reached significance, with unadjusted p-values (significance threshold in Functional ANOVAs, *F*(3, 41) = 2.83, *p*<.05). Square brackets indicate the significant portion of the event region with adjusted p-values (*p*<.001). The same pattern of Performer-specific differences was obtained in *keypress* event regions (47% of samples) and *at-rest* regions (58%). Hence, performers' movement kinematics differed in particular in the vicinity of keypresses. A similar pattern was observed for other fingers.

The next step was to determine whether these differences reflect performer-specific kinematic signatures. Specifically, we tested whether it was possible to generate a model of finger movement kinematics for a given performer that was capable of identifying that performer on the basis of novel movement trajectories. To test this possibility, a neural network classifier was trained and then tested on the *attack*, *at-rest*, and *keypress* portions of the velocity/acceleration trajectories that differed across performers. The significant portions of the velocity and acceleration trajectories were first entered in a Principal Component Analysis, to reduce the amount of information. Five principal components, which accounted for more than 95% of the variance in the finger trajectories, were used to train a two-hidden-layer neuronal network (with 10 and 20 hidden units) with a resilient back-propagation algorithm, which yields superior classification performance in automatic pattern recognition [33]. Network classification performance was optimized using bootstrap methods (bagging methods) [34]. Five 10-fold cross-validation experiments were performed, in which data were divided into 10 subsets. The performer's identity was predicted for each subset by a neural network classifier that was trained on the remaining 9 subsets. This process was repeated 5 times. Mean classification accuracy, as reported here, is based on the average of these 5 cross-validation experiments.

**Figure 7:**
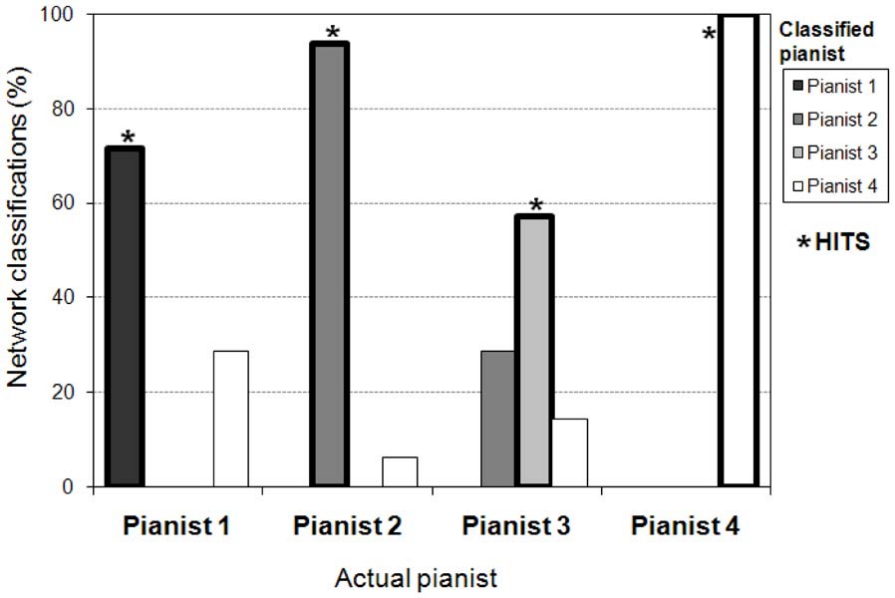
Percent neural network classifications by actual and classified pianist, based on principal components of velocity-acceleration trajectories for all finger movements in the *attack* event region. Stars indicate correct classifications or HITS (when the classified pianist corresponded to the actual pianist).

Overall, the neural network classifiers were successful in identifying performers based on finger movements in the *attack* and *keypress* event regions and were less successful when fingers were *at rest*. Figure 7 displays the network classifications by actual performer and by classified performer for the *attack* movements. The performers were correctly classified overall in 87% of *attack* trajectories (chance = 25%) and in 84% of *keypress* trajectories. Trajectories of fingers *at rest* (while another finger struck a key) were classified less successfully (76%) but better than chance (25%). The network's successful classifications of the event finger trajectories (in proportions) were entered in an ANOVA by Event region (attack, keypress, at-rest) and Performer (1–4), with trajectory as the random variable. The ANOVA confirmed the higher classification accuracy for fingers during attacks and keypresses (*F* (2, 2238) = 22.61, *p*<.01).

Also evident in Figure 7 is the fact that some performers were identified correctly more often than others (*F* (3, 2238) = 20.67, *p*<.01); finger trajectories of Performers 2 and 4 were classified correctly overall (97%) more often than those of Performers 1 and 3 (64%). Correct classifications for individual performers also differed for *attack*, *keypress*, or *at-rest* event regions, as indicated in a significant interaction (*F* (6, 2238) = 5.46, *p*<.01). Post-hoc tests (Tukey HSD) revealed that trajectories of fingers *at rest* were significantly less successful than *attacks* (*p*<.05) and *keypresses* (for performer 3 only, *p*<.01) for classifying Performers 1 and 3. Differences among event regions for Performers 2 and 4 did not reach significance.

Several characteristics of the sounded tones differed in the performances for which the network correctly classified the movement trajectories. Correctly classified trajectories in the *attack* event regions were characterized by a small but significant increase in temporal accuracy (16 ms) relative to incorrectly classified trajectories (19 ms), as indicated by the absolute difference between the performed IOI and the IOI prescribed by the metronome (*t* (448) = 2.32, *p*<.05) . Performers 2 and 4, whose finger movements were more often classified correctly, had more piano performance experience (20.5 years on average) than Performers 1 and 3 (12.0 years on average). In addition, Performers 2 and 4 displayed higher consistency of movement (higher Procrustes similarity values) than Performers 1 and 3 (*t* (147) = 2.4, *p*<.05, with trajectories as the random variable). Thus, network identification of performers based on single finger movements was more accurate for skilled performers with consistent finger movements and for movements before and after goal-related keypresses. Movement trajectories were more consistent and led to more accurate performer identification when they were temporally accurate.

Further analyses were conducted to test whether variability in movement kinematics across pianists reflected in sound differences allowed for performer classification. To this aim, MIDI event data (key velocity and tempo accuracy, expressed as before in average deviation of the performed IOI from the expected IOI, in percent) were analyzed across all tempi and the two melodies. The four performers differed in terms of key velocity (Performer 1 = mean of 60.3 MIDI units; Performer 2 = 61.5; Performer 3 = 33.9; Performer 4 = 48.0; *F*(3, 446) = 122.95, *p*<.001) and tempo accuracy (Performer 1 = 16.6%; Performer 2 = 8.4%; Performer 3 = 7.6%; Performer 4 = 6.6%; *F*(3, 446) = 25.36, *p*<.001). Post-hoc analyses (with Tukey HSD, *p*<.001) confirmed that each performer significantly differed from the others in terms of either key velocity or timing accuracy. Finally, in order to assess whether MIDI event data are sufficient to predict which pianist is performing, key velocities and timing accuracy were entered in a multiple regression model using pianist number as the dependent variable. The analysis showed that the model significantly predicted the performer (*r-squared* = .23, *F*(2, 447) = 67.78, *p*<.001); moreover, both key velocities (*β* = −.39, *p*<.001) and timing accuracy (*β* = −.21, *p*<.001) significantly contributed beyond the other variable to explain variance. In sum, these results confirm that both finger kinematics and the sounded result of movement consistently allowed for pianist classification.

## Discussion

Kinematic and acoustic measures of pianists' performances yielded two novel findings. First, faster performances yielded greater finger heights above the keys, which were related to larger key velocities; finger kinematics in the vicinity of peak heights contain relevant information for differentiating performers. Peak heights consistently occurred about one tone before keypresses. This finding contradicts both empirical findings in simple finger movement tasks and pedagogical recommendations for music performance practice. Second, individual pianists' finger movements were characterized by unique velocity/acceleration patterns of goal-directed movements toward a keypress. The specificity of these kinematic patterns was demonstrated by successful network classifications of performers, based on trajectories in the *attack* and *keypress* events as compared to *at-rest* portions of performance.

Previous measures of finger coordination between hands indicated smaller finger movement amplitudes at faster tempi [24]. In addition, some pedagogical techniques recommend that musicians reduce their finger movement at faster tempi, in order to conserve energy [22]. Yet, this finding is consistent with recent evidence in duet piano playing showing a rate effect for finger amplitudes: the fingers were raised higher above the keyboard when pianists had to play eighth notes as compared to quarter notes [35].

The observed increase of finger movement amplitude with tempo is unlikely to result solely from differences in performance style or expression, for the simple melodies recorded here. The effects of tempo on finger height were still present when differences in key velocity associated with increased loudness were partialled out (see [36] for other evidence confirming that intensity goals are not the sole cause of increased finger height at fast tempi). As well, clarinetists have been shown to raise their fingers farther above clarinet keys at faster tempi [37], despite a lack of change in accompanying sound intensity. Additionally, Loehr and Palmer [5] showed that pianists' finger taps increased in height as the tempo increased, and pianists' increased finger heights were accompanied by increased finger-key contact accelerations, which correlated with better temporal precision of upcoming keypresses [16]. These findings suggest that sensorimotor integration, rather than style or expressive goals, accounts for the need to raise fingers higher at faster tempi. More likely, the tendency of pianists' finger heights to increase with tempo may be related to goals of spatial and temporal precision in music performance, which differ from traditional motor tasks. Most motor studies employ single-effector movements whose goal is spatial accuracy [38], [39], [40]. Some tasks require repetitive movements in which participants focus either on spatial accuracy, such as reaching tasks [41] or temporal accuracy, such as tapping tasks [5], [42]. Piano performance requires both spatial and temporal accuracy, involves more than two effectors, and its ultimate goal is sound production.

Larger amplitudes of motion at faster tempi may partially compensate for the observed trade-off between speed (tempo) and accuracy (spatial and temporal accuracy) [41]. Fast tempi in piano performance usually entail lower spatial accuracy (more wrong keys pressed; [1], [2]) and higher temporal variability [17], [18], [19]. Skilled performers may adopt movement strategies (e.g., increasing movement amplitude) aimed at containing the deleterious effects of speed on spatial and temporal accuracy at very fast tempi, in order to achieve error-free performances. This possibility is consistent with recent observations that changes in pianists' finger kinematics have a direct effect on temporal accuracy. Greater finger acceleration when pianists' fingers make contact with the key surface predicts temporal accuracy for the temporal interval following the keystroke: the larger the change in acceleration at finger-key contact, coincident with greater tactile and kinesthetic feedback, the more accurate the subsequent temporal interval produced by pianists [16]. Strategies such as those aimed at increasing movement accuracy, thereby compensating for a speed-accuracy trade-off, may have played a role in the present study. A speed-accuracy tradeoff is the tendency toward lower accuracy (e.g., in error rates or temporal variability) at faster tempi. This constraint is obviously incompatible with one of the main goals of piano performance, namely to maintain high temporal accuracy at fast tempi. Pianists may have increased movement amplitude at faster tempi, thus enhancing temporal accuracy to counter a speed-accuracy tradeoff. Related to this is the idea that effects of tempo on movement amplitude were guided by perceptual factors. By increasing finger movement amplitude, pianists may have increased tactile and kinesthetic feedback at keypress, to overcome the decrease in temporal accuracy that typically occurs at faster tempi (in accordance with Weber's law). This explanation, although plausible, awaits further inquiry. For example, sensory properties were not manipulated in the present study, a possibility that deserves further research.

Finally, pianists' finger movements demonstrated consistent kinematic differences in velocity/acceleration patterns across fingers and melodies, in goal-directed movements in the vicinity of keypresses. The specificity of these kinematic patterns was demonstrated by highly successful network classifications of performers based on trajectories in the *attack* and *keypress* event regions as compared to *at-rest* regions. Success rate in classifying trajectories was also sensitive to sounded differences among pianists, and to pianists' performance experience. Nonetheless, movement differences contributed to classification success beyond these factors. It is striking that so little kinematic information was sufficient to extract a model of performers' individualistic movement signatures that allowed successful classification of new trajectories; this finding is significant, considering that pianists were not chosen *a priori* as having different performance styles. One caveat is that the large number of individual keypresses examined here represented a small number of pianists and a small number of melodies. A larger sample is needed to generalize these findings to other performance situations. In addition, the present study did not examine whether humans can classify pianists based on visual information. However, such study should be conducted in the future to confirm the performance of the neural network classifier.

Subtle individual movement differences also emerge in handwriting, typing, and other finger actions [43]. Increasing evidence suggests that personal identifiers may be found in many goal-directed actions [44], [45], [46]. The fact that finger movements in piano performance give rise to specific sound characteristics suggests that what makes performers unique begins with their movements. Individual characteristics of finger movements can have ramifications for pianists' touch that influence their control of force and timing [16], which in turn yields individualistic sound.

## Method

### Ethics statement

Written informed consent, as required by the ethics committee of the Ohio-State University, was obtained from all participants.

### Participants

Four skilled adult pianists (mean age = 24 years, range = 18–40 years, 3 females and 1 male) with an average of 16.3 years of piano performing experience (range 12–21 years) were recruited from the Columbus, Ohio, music community. Performers 1 and 3 had less piano performance experience (12.0 years on average) than Performers 2 and 4 (20.5 years on average). There were no large anatomical differences in hand size or shape between participants. Participants received a nominal fee for taking part in the experiment.

### Stimulus Materials and Equipment

**Figure 8:**
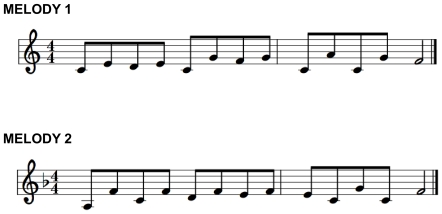
Melodies used in the experiment.

Two simple 4-measure isochronous melodies notated in 4/4 meter (one in C-major, one in F-major), each containing 13 notes for the right hand, were created for the experiment (see Figure 8). The melodies were constructed such that horizontal hand displacement was limited and no “thumb-under” movement was required, to limit occlusions during motion capture.

Participants performed the melodies on a Roland RD600 MIDI digital piano. Finger movements were recorded by a Vicon-8 motion capture system (Vicon Motion Systems Ltd.). Fourteen cameras with fine-angle lenses, located around the pianist, captured the movement of passive markers (3-mm diameter) glued to the fingernails on the right hand (sampling frequency = 120 Hz, yielding 8-ms temporal resolution, which was about 7% of the interonset intervals (IOIs) at the fastest prescribed tempo; spatial resolution = .01 mm). The markers did not interfere with fingers' movement during piano performance. Additional markers were placed on the finger joints and on the front surface of the piano keys (one for each key), to align the movement trajectories with the MIDI data at the pofint of minimum key position. Sonar 2.2 software was used to record MIDI keypress timing data (“note on” times) from the digital piano.

### Procedure

The pianists first completed a questionnaire on their musical background, and then memorized each melody, using their own choice of fingering, until they could play it from memory without errors. Three of the participants adopted the same fingering (for melody 1, Pianists 1–3 used the fingers 1 3 2 3 1 5 4 5 1 5 1 4 3; for melody 2, Pianists 2–4 used the fingers 1 5 2 5 3 5 4 5 3 1 5 1 4). The other participant adopted a slightly different choice of fingering, differing by three events in melody 1, and by one event in melody 2. Pianists then performed each melody from memory, following the tempo indicated by a metronome at the quarter-note level. Each melody was performed at five experimental tempi in the following order, progressively from the easiest to the most difficult tempo: Slow tempo (60 beats/min, eighth-note IOI = 500 ms), Fast1 (180 beats/min, eighth-note IOI = 167 ms), Fast2 (210 beats/min, eighth-note IOI = 143 ms), Fast3 (240 beats/min, eighth-note IOI = 125 ms), and Fast4 (250 beats/min, eighth-note IOI = 120 ms). The wide range of fast tempi was chosen to induce a broad range of possible finger movements under conditions that required fast preparation. The metronome sounded prior to each performance and was turned off while the performance was recorded. Each pianist performed each melody at each tempo at least once; only error-free performances, assessed by comparing the performances with the notation, were analyzed, totaling 45 performances. All pianists were able to provide errorless performances at Slow (*n* = 14) and Fast1 tempi (*n* = 13). Three performers were able to perform the melodies at the Fast2 tempo (*n* = 11); two performers at Fast3 tempo (*n* = 5), and one performer at the Fast4 tempo (*n* = 2). All performances of one melody by one performer were discarded due to inadvertent hand-rubbing that caused marker displacement. The experimental session lasted approximately one hour.
